# Exploring Social Cognition Sub‐Domains and Predictors in Multiple Sclerosis: A Cross‐Sectional Study

**DOI:** 10.1002/brb3.70691

**Published:** 2025-07-21

**Authors:** Ozlem Totuk, Merve Turkkol, Ebru Hatun Uludaşdemir, Hasan Can Güdek, Guldeniz Cetin Erci, Ipek Gungor Dogan, Damla Cetinkaya Tezer, Sevki Sahin, Serkan Demir

**Affiliations:** ^1^ Hamidiye Faculty of Medicine, Department of Neurology, Sancaktepe Prof. Dr. Ilhan Varank SUAM University of Health Sciences Istanbul Türkiye

**Keywords:** cognitive impairment, emotion recognition, multiple sclerosis, social cognition, social intelligence

## Abstract

**Background and purpose:**

Social cognition (SC) is increasingly recognized as a key cognitive domain affected in multiple sclerosis (MS), yet its sub‐domains and clinical correlates remain underexplored. This study aimed to assess different SC sub‐domains and identify their cognitive, emotional, and demographic predictors in people with MS (pwMS).

**Methods:**

This cross‐sectional study included 93 pwMS and 34 HCs. Assessments included the Reading the Mind in the Eyes Test (RMET) for emotion recognition, the Trail Making Test (TMT) for executive function, the Tromso Social Intelligence Scale (TSIS) for nonverbal understanding, the Implied Meaning Test (IMT) for implicit understanding, the Social‐Emotional Competence Scale for adaptability, the Barratt Impulsiveness Scale for impulsivity, the Stroop Test for inhibition, the Beck Depression Inventory (BDI) for depression, the Montreal Cognitive Assessment (MoCA) for cognition, and the Short Form‐12 (SF‐12) for quality of life (QoL). Multiple regression analyses were conducted to identify independent predictors of SC performance.

**Results:**

PwMS, particularly those with progressive MS, exhibited significantly lower SC performance across all sub‐domains compared to HCs. Regression analyses revealed that lower MoCA scores, higher BDI scores, and lower educational attainment were significant predictors of impaired SC, while disease duration and gender were not. Notably, SC deficits were also observed in cognitively preserved individuals, suggesting the relative independence of SC impairments.

**Conclusion:**

SC impairment is a distinct and clinically relevant feature of MS, associated with both cognitive and emotional factors. Routine SC screening may enhance patient care by informing personalized interventions. Future research should include larger cohorts, longitudinal designs, and practical SC assessment tools for clinical use.

## Graphical abstract

This graphical abstract illustrates the associations between social cognition sub‐domains (emotion recognition, social intelligence, adaptability, and implicit understanding) and their cognitive, emotional, and demorgraphic predictors in people with multiple sclerosis. Progressive MS patients showed the most pronounced impairments. Lower global cognition, higher depression scores, and lower educational attainment were significant predictors of poor social cognition.

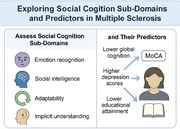

## 1 Introduction

Multiple sclerosis (MS) is a chronic, immune‐mediated inflammatory disease characterized by inflammation, demyelination, and axonal loss, primarily affecting the central nervous system (CNS) (Miller et al. 2018). It is one of the leading causes of non‐traumatic disability in young adults and is associated with both physical and cognitive impairments (Miller et al. 2018). Cognitive deficits in MS commonly involve impairments in information processing speed, attention, memory, and executive functioning (Miller et al. 2018; Frith 2008). In addition to these well‐documented cognitive challenges, social cognitive impairments (SCIs) have gained increasing attention due to their significant impact on quality of life (QoL) and social functioning in people with MS (pwMS) (Dulau et al. 2017).

Social cognition (SC) refers to the capacity to perceive, interpret, and respond appropriately to social stimuli, including others' emotions, thoughts, and intentions (Dulau et al. 2017; Henry et al. 2016). These abilities are essential for daily interpersonal interactions and encompass empathy, social judgment, and emotional recognition (Henry et al. 2016; Halstead et al. 2021). Emerging evidence suggests that SCIs may manifest early in the disease course and progressively worsen; however, their associations with disease severity, cognitive function, and psychosocial outcomes remain insufficiently explored (Halstead et al. 2021; Genova and McDonald 2020).

Recent studies have emphasized the importance of SCIs in MS due to their links with reduced QoL and increased social withdrawal (Genova and McDonald 2020; Henry et al. 2015). While various neuropsychological tests have been employed to assess SC in MS—such as those measuring emotion recognition, theory of mind (ToM), and empathy—many fail to provide a comprehensive evaluation of all SC domains. Additionally, prior studies often suffer from small sample sizes and methodological inconsistencies (Henry et al. 2015; Thompson et al. 2018; Kurtzke 1983).

The present study aims to compare SC subcomponents between pwMS and healthy controls (HCs), stratified by MS subtype; examine associations between SCIs, cognitive function, depression, and QoL; and identify brief and clinically applicable tools for SC assessment. Early identification and intervention for SCIs may help prevent deterioration in social communication and improve QoL in pwMS.

We hypothesized that individuals with MS, particularly those with progressive subtypes, would exhibit significant SC deficits compared to HCs and these deficits would correlate with cognitive impairment, depressive symptoms, and decreased QoL. Evaluating SC in MS not only enhances our understanding of cognitive decline related to the disease but also carries clinical importance. Timely identification of SCIs can guide individualized care, support targeted interventions, and inform rehabilitation strategies to improve social functioning and overall well‐being.

## 2 Materials and Methods

### 2.1 Participants

This cross‐sectional study was approved by the local ethics committee and conducted in accordance with the Declaration of Helsinki. Participants were recruited from the MS outpatient clinic of Sancaktepe Prof. Dr. İlhan Varank Training and Research Hospital between November 2023 and May 2024. The study sample comprised 93 patients with MS, diagnosed with either relapsing‐remitting MS (RRMS) or progressive MS (PMS, including both primary and secondary forms), according to the revised McDonald criteria (Thompson et al. 2018). A control group of 34 healthy individuals with no history of CNS disorders was also included. HCs were matched to the MS group by age and educational level, and statistical analyses confirmed no significant differences between the groups in these variables.

Written informed consent was obtained from all participants before enrollment. MS diagnoses, clinical subtype classifications, and clinical follow‐up evaluations were conducted by a neurologist in the MS outpatient clinic.

The functional status of pwMS was assessed using the Expanded Disability Status Scale (EDSS) (Kurtzke 1983). Demographic data, disease duration, EDSS scores, and current treatments were recorded for each patient. Educational level was quantified based on total years of formal education (e.g., 12 years for high school, 16 years for university).

Patients were excluded if they had experienced a clinical relapse or received corticosteroid treatment within the preceding 3 months to minimize potential confounding effects of acute disease activity or treatment.

All participants (pwMS and HCs) completed a comprehensive battery of SC assessments. In addition, only pwMS were evaluated using the Montreal Cognitive Assessment (MoCA) for global cognition, the Beck Depression Inventory (BDI) for depressive symptoms, and the Short Form‐12 (SF‐12) for health‐related QoL. All assessments were administered individually by a trained neuropsychologist in a quiet, well‐lit environment in a single session lasting approximately 60–90 min.

A post hoc power analysis using G*Power version 3.1 revealed an achieved power (1–β) of 0.85 for detecting medium effect sizes (Cohen's *d* = 0.5) in between‐group comparisons, confirming the adequacy of the sample size for the primary analyses.

### 2.2 Measurement Tools

Below is a brief overview of the assessment instruments used in this study, focusing on their relevance to the study aims and their role in evaluating SC and related domains among individuals with MS.

#### 2.2.1 Beck Depression Inventory

The BDI is a 21‐item self‐report scale developed by Beck et al. (1961) to assess emotional, cognitive, and somatic symptoms of depression. Higher scores reflect more severe depressive symptoms. The Turkish version, validated by Hisli (1998), considers scores ≥ 17 as indicative of clinically significant depression and reports a Cronbach's alpha of 0.80.

#### 2.2.2 Reading the Mind in the Eyes Test (RMET)

Developed by Baron‐Cohen et al. (2001), the RMET evaluates the ability to recognize emotions by presenting photographs of eye regions and requiring participants to identify the most appropriate mental state from four options. The Turkish adaptation by Yıldırım et al. (2011) demonstrated acceptable reliability (Cronbach's alpha = 0.71).

#### 2.2.3 Trail Making Test (TMT)

The TMT assesses visual attention, processing speed, and task‐switching abilities. It consists of two parts (A and B), with Part B imposing greater executive demands (Reitan 1958). The Turkish standardization by Cangoz et al. (2007) reported a Cronbach's alpha of 0.73 for Part B.

#### 2.2.4 Tromsø Social Intelligence Scale (TSIS)

The TSIS measures social intelligence across three subdomains: social information processing, social skills, and social awareness. It includes 21 items rated on a 7‐point Likert scale (Silvera et al. 2001). The Turkish version by Doğan and Çetin (2009) demonstrated high internal consistency (Cronbach's alpha = 0.88).

#### 2.2.5 Implied Meaning Test (IMT)

The IMT, developed by Corcoran et al. (1995), assesses the ability to infer implied meanings from short narratives. Cronbach's alpha values range from 0.73.

#### 2.2.6 Social Emotional Competence Scale (SECS)

The SECS is a 25‐item self‐report instrument evaluating two key dimensions of social‐emotional competence: adaptability and expressiveness (McBrien et al. 2020). The original version reported Cronbach's alpha values ranging from 0.73 to 0.86.

#### 2.2.7 Barratt Impulsiveness Scale (BIS)

The BIS is a self‐report measure used to assess impulsivity. Higher scores indicate greater impulsiveness (Steinberg et al. 2013). The Turkish adaptation by Güleç et al. (2008) reported good internal consistency (Cronbach's alpha = 0.82).

#### 2.2.8 Stroop Test (TBAG Form)

Originally designed by Stroop (1935), this test assesses cognitive flexibility and resistance to interference. The Turkish‐adapted TBAG form used in this study demonstrated high reliability (Cronbach's alpha = 0.86) (Karakaş et al. 1999).

#### 2.2.9 Montreal Cognitive Assessment

The MoCA is a widely used screening tool for cognitive function, evaluating domains such as memory, executive functioning, attention, language, and orientation (Nasreddine et al. 2005). The Turkish adaptation by Ozdilek and Kenangil (2014) considered scores ≥ 21 as cognitively normal, with a Cronbach's alpha of 0.83.

#### 2.2.10 Short Form‐12

The SF‐12 is a condensed version of the SF‐36, designed to assess physical and mental components of health‐related QoL (Ware and Sherbourne 1992). The Turkish version, validated by Kocyigit (1999), demonstrated strong reliability with Cronbach's alpha values of 0.88.

### 2.3 Statistical Analysis

Statistical analyses were performed using SPSS version 27 (IBM Corp 2020) and R version 4.3.0 (R Core Team 2023). Descriptive statistics and frequency distributions were first calculated to summarize the demographic and clinical characteristics of the participants.

The normality of continuous variables was assessed using both skewness and kurtosis values (acceptable range: −3 to +3) (Kline 2011), in addition to the Shapiro–Wilk test (Shapiro and Wilk 1965).

Between‐group comparisons were conducted using independent samples *t*‐tests (for two groups), and either one‐way ANOVA or Welch's ANOVA (for three or more groups) depending on the assumption of variance homogeneity. Post hoc analyses were carried out using either Tukey's test (for equal variances) or Tamhane's T2 test (for unequal variances).

To control for Type I error due to multiple comparisons, appropriate post hoc corrections were applied.

Effect sizes were calculated as Hedges' g (with 95% confidence intervals) for two‐group comparisons and eta squared (*η* ^2^) for multi‐group comparisons. Effect sizes were interpreted based on established conventions: small (*g* ≈ 0.20, *η* ^2^ ≈ 0.01), medium (*g* ≈ 0.50, *η* ^2^ ≈ 0.06), and large (*g* ≥ 0.80, *η* ^2^ ≥ 0.14) (Cohen 1988; Hedges and Olkin 1985; Lakens 2013).

Pearson correlation coefficients were used to evaluate associations between normally distributed variables. Prior to analysis, assumptions of linearity and homoscedasticity were visually inspected using scatterplots. Correlation matrices were visualized using the R package “GGCorplot” (Kassambara 2019). Pearson correlation analysis was only performed for variables that met the assumption of normality. Assumptions of linearity and homoscedasticity were assessed through visual inspection of scatterplots. The analysis was conducted using the GGCorplot package in R (version 4.3.0), and 95% confidence intervals were reported where relevant.

A multiple linear regression analysis was conducted to identify predictors of SC, with the Tromsø Social Intelligence Scale (TSIS) score used as the dependent variable. Predictor variables included demographic factors (age, sex, and education), cognitive performance (MoCA scores), affective status (BDI scores), and impulsivity (BIS subscale scores).

Normality of residuals was verified using both skewness/kurtosis criteria and the Shapiro–Wilk test. Multicollinearity was checked via variance inflation factors (VIF).

## 3 Results

Table 1 presents the demographic characteristics of the study participants. Of the total sample, 60.6% were women, with 51.2% diagnosed with RRMS, 22.0% with PMS, and 26.8% identified as HCs. More than half of the MS patients had been living with the disease for over 3 years. The most frequently used medication was Ocrelizumab (41.9%). The mean age of the participants was 35.8 years (SD = 10.17).

**TABLE 1:** Demographic characteristics of participants.

Variable	Group	*n*	%
**Gender**	Female	77	60.6
	Male	50	39.4
**Diagnosis**	RRMS	65	51.2
	Progressive	28	22.0
	Healthy control	34	26.8
**Disease duration**	< 1 year	30	32.3
	1–3 years	12	12.9
	≥ 3 years	51	54.8
**EDDS score**		2.610 ± 1.710	
**Medication**	Low/moderate efficacy DMT	25	26.9
	High efficacy DMT	53	57.0
	Symptomatic/no DMT	15	16.1
**Age**		35.833 ± 10.170	

*Note*: The low/moderate efficacy DMT (first‐line) group includes parenteral Interferon‐β derivatives (Rebif, Betaferon, Avonex, and Peg‐Interferon) and Glatiramer Acetate (Copaxone), as well as oral agents such as Dimethyl Fumarate (Tecfidera, Difurat, Pharon, Lidvina, and Tenipra) and Teriflunomide (Aubagio and Teflimes); these treatments have similar, moderate disease‐modifying effects. The high efficacy DMT group includes immunosuppressive/restructuring agents such as fingolimod (Vintor, Fingya, Judexa, Finimod, and Gilomid), ocrelizumab (Ocrevus), rituximab (MabThera and off‐label), and cladribine (Mavenclad). The symptomatic/no DMT group includes only participants using Fampridine (Fampyra), which is intended to improve walking speed, and those not receiving any disease‐modifying therapy.

Abbreviation: *n*, number of observations.

Table 2 presents the comparison results of SC and psychometric scores among the RRMS, PMS, and HC groups.

**TABLE 2:** Comparisons of social cognition and psychometric measurements of RRMS, progressive MS, and healthy control groups.

Variable/group	RRMS mean ± SD	Progressive mean ± SD	Healthy control mean ± SD	*t*	*p*	*η* ^2^	95% CI (*η* ^2^)
Reading the mind in the eyes test–total score	23.57 ± 4.28	22.43 ± 2.44	27.18 ± 2.96	7.466	< 0.001*	0.546	0.435–0.670
Social emotional competence–adaptability	60.42 ± 11.11	62.96 ± 5.40	60.47 ± 10.16	1.526	0.224	0.024	0.000–0.106
Social emotional competence–expressivity	32.18 ± 6.45	33.86 ± 3.39	37.11 ± 5.79	1.549	0.247	0.026	0.000–0.108
Tromso social intelligence scale–total score	71.08 ± 9.46	69.43 ± 10.77	76.88 ± 8.62	5.884	< 0.001*	0.480	0.359–0.607
Barratt–non‐planning	9.88 ± 3.46	14.25 ± 3.96	9.09 ± 2.14	2.4	0.119	0.112	0.063–0.298
Barratt–motor impulsivity	8.39 ± 3.15	10.14 ± 3.65	6.78 ± 2.03	2.4	0.115	0.113	0.053–0.288
Barratt–attentional impulsivity	8.87 ± 3.44	11.39 ± 4.02	7.65 ± 2.55	3.6	0.001*	0.173	0.096–0.348
Barratt–total score	27.13 ± 6.46	41.39 ± 10.67	23.53 ± 5.65	5.55	< 0.001*	0.181	0.102–0.346
IMT–total score	7.14 ± 0.96	—	7.79 ± 0.75	—	—	—	—
EDSS score	1.93 ± 1.42	4.31 ± 1.08	—	8.66	< 0.001*	0.394	0.293–0.556
SF‐12 score	38.45 ± 5.62	32.93 ± 6.32	41.82 ± 4.87	7.312	< 0.001*	0.373	0.273–0.540
MOCA test score	25.11 ± 2.97	22.18 ± 2.70	27.86 ± 2.78	1.78	0.275	0.033	0.000–0.154
Beck depression score	12.23 ± 7.56	10.32 ± 2.79	6.00 ± 1.94	1.94	< 0.001*	0.372	0.250–0.519

*Note*: values marked as “–” could not be calculated due to the absence of observations in the respective groups. Additionally, statistically significant differences between groups are presented using superscript letters. According to multiple comparison results, groups sharing the same letter do not differ significantly, whereas those with different letters show significant differences. f, independent samples ANOVA test; mean, average; t, independent samples *t*‐test; w, Welch test. *η* ^2^: ANOVA/Welch ANOVA effect size.

Abbreviations: CI, confidence interval; SD, standard deviation.

No statistically significant differences were found among the groups in the SECS adaptability and expressiveness subscales (*p* > 0.05). However, participants with RRMS (*M* = 23.57, SD = 4.28) and PMS (*M* = 22.43, SD = 2.44) scored significantly lower on the RMET than HCs (*M* = 27.18, SD = 2.96), Welch *F*(2, *x*) = 74.618, *p* < 0.001, with a large effect size (*η* ^2^ = 0.546, 95% CI [0.435, 0.670]).

TSIS scores were significantly lower in the PMS group (*M* = 43.93, SD = 7.09) compared to both RRMS (*M* = 71.02, SD = 15.49) and HCs (*M* = 69.76, SD = 18.84), Welch *F* = 78.447, *p* < 0.001, *η* ^2^ = 0.559, 95% CI [0.449, 0.680].

Participants with PMS scored significantly higher on the BIS Non‐Planning (*M* = 14.25, SD = 5.45), Attentional Impulsivity (*M* = 14.14, SD = 5.62), and BIS Total (*M* = 41.39, SD = 16.27) subscales compared to both RRMS and HC groups (*p* < 0.001), indicating medium effect sizes (*η* ^2^ = 0.159–0.202). Motor impulsivity scores were also significantly higher in the PMS group compared to HCs (*p* < 0.05).

IMT scores were significantly higher in the HC group (*M* = 7.79, SD = 0.73) than in the RRMS (*M* = 7.14, SD = 0.95) and PMS (*M* = 6.79, SD = 0.92) groups, Welch *F* = 12.986, *p* < 0.001, *η* ^2^ = 0.173. PMS patients had significantly higher EDSS scores and lower SF‐12 scores than the RRMS group (*p* < 0.05).

MoCA scores declined significantly in the order of HC > RRMS > PMS (*p* < 0.05), with mean scores of 27.76 (SD = 1.78), 25.11 (SD = 2.97), and 22.18 (SD = 2.78), respectively. Welch *F* = 45.723, *p* < 0.001, *η* ^2^ = 0.424. HCs had significantly lower BDI scores compared to RRMS and PMS groups (*p* < 0.05), indicating higher depressive symptom burden in pwMS. SF‐12 scores were also significantly lower in the PMS group (*M* = 31.57, SD = 4.80) than in the RRMS group (*M* = 38.45, SD = 5.28), Welch *t* = 33.857, *p* < 0.001, *η* ^2^ = 0.926.

Table 3 presents the comparison results of psychological and cognitive measures by gender among the RRMS, progressive MS, and healthy control groups.

**TABLE 3:** Comparison of RRMS, progressive MS and healthy control diagnostic groups with psychological and cognitive measurements by gender.

Variable/group	RRMS and progressive (*n* = 93)				*t*	*P^t^*	*g*	95% Cl	Healthy control (*n* = 34)				t	P^t^	g	95% Cl
	Female (*n* = 55)		Male (*n* = 38)						Female (*n* = 22)		Erkek (*n* = 12)					
	Mean	SD	Mean	SD					Mean	SD	Mean	SD				
Reading the mind in the eyes test–total Score	23.11	4.29	23.39	3.12	−0.351	0.726	−0.07	−0.49; 0.35	27.86	3.27	25.92	1.78	1.905	0.066	0.67	−0.09; 1.42
Social emotional competence–adaptability	60.25	11.82	62.53	5.35	−1.252	0.214	−0.23	−0.65; 0.19	58.91	9.87	63.33	10.49	−1.222	0.230	−0.43	−1.17; 0.31
Social emotional competence–expressivity	31.69	6.52	34.13	4.03	−2.227	**0.028**	−0.43	−0.85; −0.01	32.41	5.63	36.08	4.87	−1.903	0.066	−0.67	−1.42; 0.08
Tromsø social intelligence scale–total score	67.56	15.72	56.05	19.97	2.973	**0.004**	0.65	0.22; 1.08	67.32	18.89	74.25	18.71	−1.026	0.313	−0.36	−1.10; 0.38
Stroop–interference time (seconds)	39.84	12.17	39.63	17.2	0.067	0.947	0.01	−0.40; 0.43	34.59	8.78	34.75	10.91	−0.046	0.963	−0.02	−0.75; 0.71
Stroop–number of errors	1.27	2.06	1.39	1.7	−0.301	0.764	−0.06	−0.48; 0.36	0.18	0.5	0.17	0.39	0.091	0.928	0.02	−0.71; 0.75
TMT‐B minus A difference	35.6	9.95	37.03	21.67	−0.379	0.706	−0.09	−0.51; 0.33	29.41	8.62	26.92	8.1	0.822	0.417	0.29	−0.45; 1.02
Barratt–non‐planning	10.38	4.08	10.84	5.71	−0.454	0.651	−0.09	−0.51; 0.32	9.05	2.44	9.17	2.44	−0.138	0.891	−0.05	−0.78; 0.68
Barratt–motor impulsivity	10.82	9.69	9.92	5.13	0.522	0.603	0.11	−0.31; 0.53	8.55	1.84	8.58	2.61	−0.049	0.961	−0.01	−0.74; 0.72
Barratt–attentional impulsivity	10.04	4.36	10.66	5.62	−0.6	0.55	−0.13	−0.54; 0.29	8.18	1.87	7.42	1.78	1.16	0.255	0.40	−0.33; 1.14
Barratt–total score	31.89	14.52	31.18	16.03	0.221	0.825	0.05	−0.37; 0.47	25.73	5.07	25.5	6.57	0.112	0.911	0.04	−0.69; 0.77
IMT–total score	7.07	1	6.97	0.88	0.493	0.624	0.1	−0.32; 0.52	7.95	0.21	7.5	1.17	1.336	0.208	0.62	−0.13; 1.37
MOCA test score	24.35	3.29	24.05	3.1	0.432	0.667	0.09	−0.33; 0.51	27.77	1.63	27.75	2.09	0.035	0.972	0.01	−0.72; 0.74
Beck depression score	13.33	7.63	9.24	3.36	3.513	**0.001**	0.65	0.22; 1.08	6.14	2.05	5.75	1.76	0.549	0.586	0.19	−0.54; 0.93

*Note*: M: mean; t: independent samples *t*‐test.

Abbreviation: SD, standard deviation.

Significant if p<.05

### 3.1 Gender Differences in Social Cognition and Cognitive Outcomes

Among MS participants (RRMS and PMS), female patients scored significantly higher on the TSIS and BDI but lower on the SECS Expressiveness subscale compared to males (*p* < 0.05), with large effect sizes (*g* = 0.65). No significant gender differences were observed among HCs in any psychological or cognitive measure.

Table 4 presents the comparison results of psychological and cognitive measures based on age and education level among the RRMS, PMS, and HC groups.

**TABLE 4:** Comparison of psychological and cognitive measures by age and education in RRMS, PMS, and HC groups.

Variable	Value	RRMS and progressive (*n* = 93)		Healthy control (*n* = 34)	
		Age	Education	Age	Education
Reading the mind in the eyes test ‐ total score	*r*	−0.150	0.321**	−0.042	−0.247
	*p*	0.153	**0.002**	0.811	0.160
Social emotional competence ‐ adaptability	*r*	−0.037	0.015	−0.033	0.030
	*p*	0.727	0.886	0.851	0.865
Social emotional competence ‐ expressivity	*r*	−0.075	−0.027	−0.102	0.058
	*p*	0.480	0.796	0.566	0.746
Tromsø social intelligence scale ‐ total score	*r*	−0.495**	0.340**	−0.013	−0.041
	*p*	**< 0.001**	**0.001**	0.943	0.816
Stroop ‐ interference time (seconds)	*r*	0.228*	−0.208*	0.213	−0.296
	*p*	**0.029**	**0.046**	0.226	0.089
Stroop ‐ number of errors	*r*	0.250*	−0.269**	0.588**	−0.528**
	*p*	**0.016**	**0.010**	**< 0.001**	**0.001**
TMT‐B minus A difference	*r*	0.090	−0.108	0.220	−0.169
	*p*	0.392	0.305	**0.211**	0.338
Barratt ‐ non‐planning	*r*	0.402**	−0.268**	0.317	−0.384*
	*p*	**< 0.001**	**0.010**	0.068	**0.025**
Barratt ‐ motor impulsivity	*r*	0.215*	−0.081	0.127	−0.110
	*p*	**0.040**	0.444	0.474	0.536
Barratt ‐ attentional impulsivity	*r*	0.427**	−0.204	−0.155	0.065
	*p*	**< 0.001**	0.051	0.382	0.713
Barratt ‐ total score	*r*	0.357**	−0.163	0.120	−0.208
	*p*	**< 0.001**	0.120	0.498	0.238
IMT‐ total score	*r*	−0.173	0.224*	−0.216	0.132
	*p*	0.099	**0.032**	0.221	0.456
Beck depression score	*r*	−0.141	−0.114	−0.038	−0.126
	*p*	0.182	0.279	0.831	0.478
SF‐12 score	*r*	−0.555**	0.212*	—	—
	*p*	**< 0.001**	**0.043**	—	—
MOCA test score	*r*	−0.483**	0.446**	0.062	−0.145
	*p*	**< 0.001**	**< 0.001**	0.729	0.412

*p < 0.05 and **p < 0.001.

Significant if p<.05

#### 3.1.1 Age and Education Effects on Social Cognition and Cognitive Outcomes

In the MS group, age was moderately negatively correlated with TSIS (*r* = −0.495, *p* < 0.001), MoCA (*r* = −0.483, *p* < 0.001), and SF‐12 scores (*r* = −0.555, *p* < 0.001), and positively correlated with Stroop interference time (*r* = 0.228, *p* = 0.029) and BIS subscales. This suggests that older age is associated with greater cognitive and social impairment.

Educational level was weakly to moderately positively correlated with RMET (*r* = 0.321, *p* = 0.002), TSIS (*r* = 0.340, *p* = 0.001), IMT (*r* = 0.224, *p* = 0.032), MoCA (*r* = 0.446, *p* < 0.001), and SF‐12 scores (*r* = 0.212, *p* = 0.043), and negatively correlated with Stroop scores and BIS Non‐Planning, indicating a protective effect.

Among HCs, fewer significant correlations were found. However, Stroop error count was positively associated with age (*r* = 0.588, *p* < 0.001), and marital status was negatively correlated with Stroop error count and BIS Non‐Planning scores.

Although disease duration was included in the dataset and explored, statistical analysis did not reveal any significant associations between disease duration and SC scores.

**FIGURE 1:**
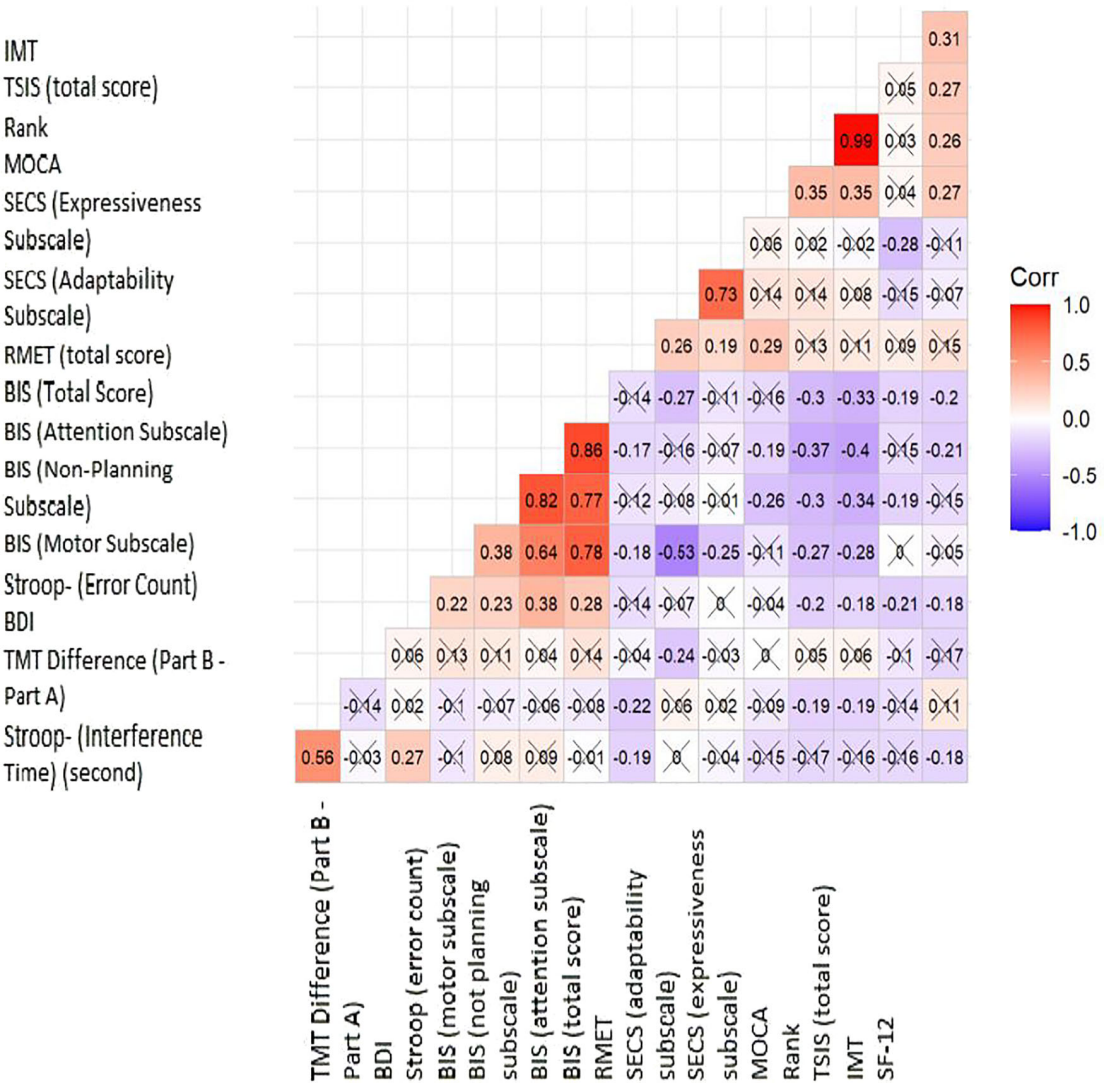
Correlations of psychological and cognitive measurements of participants in the RRMS and progressive diagnosis group.

Figure 1 presents the correlation findings of psychological and cognitive measures among participants in the RRMS and PMS groups.

### 3.2 Correlational Analysis of Social Cognition Measures

Correlation analyses revealed a weak positive correlation between RMET and both SECS adaptability (*r* = 0.252, *p* < 0.05) and MoCA scores (*r* = 0.221, *p* < 0.05), and a weak negative correlation with TMT difference scores (*r* = −0.201, *p* < 0.05).

TSIS scores were negatively associated with BIS Non‐Planning (*r* = −0.229), Motor (*r* = −0.198), and Attentional Impulsivity (*r* = −0.204), and positively associated with MoCA (*r* = 0.218) and SF‐12 scores (*r* = 0.237), indicating that lower impulsivity and better cognitive function predict stronger social intelligence.

SECS expressiveness showed negative correlations with motor impulsivity (*r* = −0.204) and IMT (*r* = −0.187). Stroop interference time correlated positively with Stroop errors (*r* = 0.281) and TMT difference (*r* = 0.345).

IMT was positively associated with SF‐12 (*r* = 0.215), and MoCA also correlated positively with SF‐12 (*r* = 0.207), supporting the functional importance of SC.

### 3.3 Predictors of Social Cognition

A multiple linear regression analysis using TSIS as the dependent variable revealed that MoCA (*β* = 0.38, *p* < 0.001), education (*β* = 0.26, *p* = 0.01), and BDI (*β* = −0.22, *p* = 0.03) were significant predictors. The model explained 42% of the variance in TSIS scores (adjusted *R* ^2^ = 0.42, F(6,86) = 12.34, *p* < 0.001) (Table 5).

**TABLE 5:** Regression model results on the effect of demographic, cognitive, and clinical variables on tromsø social intelligence score.

Model	Independent variables	B	SE	Beta	*t*	*p*	95% CI lower	95% CI upper
Model 1	Constant	77.731	13.935		5.578	0	50.042	105.420
	Age	−646	180	−0.376	−3.591	1	−1.003	−0.288
	Male (dummy)	−10.128	3.313	−0.272	−3.057	3	−16.711	−3.544
	Education level	1.005	0.662	0.158	1.519	132	−0.309	2.320
Model 2	Constant	31.842	29.279		1.088	282	−27.027	90.711
	Age	−14	275	−0.008	−0.051	959	−0.567	0.539
	Male (dummy)	−9.111	4.356	−0.246	−2.091	42	−17.870	−0.352
	Education level	1.649	1.066	0.201	1.547	128	−0.494	3.792
	MOCA test score	1.408	0.730	0.249	1.928	60	−0.060	2.876
	EDSS Score	−4.209	1.597	−0.398	−2.636	11	−7.420	−0.998
	Beck depression score	13	0.353	0.004	0.038	970	−0.695	0.722
	IMT test–total score	−1.607	2.131	−0.086	−0.754	455	−5.892	2.679

Age, sex, and BIS subscales were not significant predictors after accounting for cognitive and emotional variables. Similarly, disease duration was included in the regression model but did not emerge as a statistically significant predictor of SC.

## 4 Discussion

This study investigated the presence of SCI in individuals with multiple sclerosis (pwMS) and explored their associations with demographic, clinical, and cognitive variables. The findings revealed significant SCI, particularly among those with progressive MS (PMS), which was associated with QoL, executive functioning, depressive symptoms, and impulsivity. These results suggest that SCI constitutes an independent and clinically meaningful domain in MS, underlining the importance of early evaluation.

SC has been recognized in DSM‐5 as one of the core neurocognitive domains, playing a critical role in everyday social interactions. SCI may emerge early in the course of MS and progressively worsen over time (Halstead et al. 2021; Genova and McDonald 2020). In our study, SCI was more prevalent and pronounced in the PMS group. These results are consistent with findings reported by Dulau et al. (2017), Genova and McDonald (2020), and (Oliveira et al. 2023). Specifically, the PMS group showed lower performance on SC measures such as RMET, TSIS, and IMT, highlighting the need for closer assessment of SC in this population. Furthermore, a study by Ciampi et al. showed that SC deficits in PMS were not always accompanied by traditional cognitive impairments, suggesting distinct neuroanatomical substrates that may underlie these changes (Ziccardi et al. 2021).

The relationship between SCI and gender has been widely discussed in the literature. In our study, female patients had higher TSIS scores and depression scores but lower SECS expressiveness scores compared to males. This is in line with findings reported by Henry et al. (Förster et al. 2018) and Ziccardi et al. (2021), suggesting sex‐based differences in social cognitive functioning. Hormonal, neurological, or social factors may underlie these differences, although further research is needed to clarify these mechanisms.

SCI is also linked not only to global cognitive performance but also to educational level. In our study, higher educational attainment was positively correlated with performance on RMET, TSIS, and IMT and negatively correlated with Stroop errors and impulsivity measures. These findings support the role of cognitive reserve as described in prior research (Bora et al. 2016). This also echoes the perspectives of (Förster et al. 2018) and McCade et al.^2011^ (Amato and Portaccio 2007) on the protective effect of education on SC. However, years of formal education may not fully capture the multidimensional nature of cognitive or social cognitive reserve, warranting more comprehensive assessment approaches.

We also observed SCI in participants whose MoCA scores were in the normal range (≥21), suggesting that SCI may occur independently of global cognitive impairment. This aligns with findings by Genova et al. (Doskas et al. 2023) and Pitteri et al. (2019), who reported SCIs even in MS patients without general cognitive dysfunction. The amygdala, which plays a key role in emotional processing, has been implicated in these findings (Pitteri et al. 2019). These results further emphasize the need for domain‐specific assessment tools such as RMET and TSIS. Furthermore, dynamic assessment methods such as those used by Genova et al. (2016) have shown that traditional test batteries may miss subtle SCI in MS patients.

The relationship between executive functions and SCI has been well documented in the literature. Our study supports this association, as better performance on SC tasks was linked with better outcomes on executive function measures such as Stroop and TMT. Specifically, lower Stroop interference times and impulsivity scores were associated with better SCI performance, underscoring the role of cognitive control processes in SC (Genova and McDonald 2020; Förster et al. 2018).

The impact of disease duration on SCI remains a matter of debate. While Ciampi et al.^2018^ (Ziccardi et al. 2021) reported no significant association, Kraemer et al. (2013) suggested a positive correlation. In our analysis, disease duration did not significantly predict SC outcomes. This may be due to variability in individual cognitive reserve, the rate of disease progression, or adaptive coping mechanisms.

The link between depression and SCI is similarly inconsistent in the literature. Although BDI scores were significantly higher in both PMS and RRMS groups compared to healthy controls, we did not observe a significant correlation between SCI and depressive symptoms. This supports the idea that SCI may develop independently of mood disturbances. Our findings are consistent with those of. (Boraet al.^2016^), who also found no direct relationship between SCI and depression.

The impact of SCI on QoL is well established. In our study, higher performance on SCI tests such as TSIS and IMT was positively associated with better SF‐12 scores, reinforcing the idea that SCI is closely related to functional independence and social engagement (Pinkham et al. 2016; Elamin et al. 2012). This is consistent with the view of Isernia et al. (2019) who emphasized the dual cognitive and affective components of Theory of Mind in MS and their relevance to long‐term psychosocial functioning (Valentina et al. 2020).

Despite these findings, several limitations must be acknowledged. The absence of a direct IQ assessment limits our ability to draw conclusions about general cognitive capacity and its relationship with SCI. Additionally, we did not assess fatigue—a symptom commonly observed in MS—which may influence both cognitive and social functioning. Future research should include fatigue and standardized IQ measures to better elucidate these associations.

In conclusion, SCI is a common and clinically relevant impairment in MS, especially in individuals with PMS. It is strongly associated with both cognitive performance and QoL, and may occur even in those with globally intact cognition. These results underscore the importance of integrating SCI assessments into routine clinical evaluations for pwMS. Early recognition and targeted rehabilitation may enhance social participation and psychosocial well‐being.

## 5 Conclusion

This study demonstrates that SCI are prevalent among pwMS, particularly those with progressive MS (PMS), and are closely associated with cognitive dysfunction, increased impulsivity, and reduced QoL. Notably, SCI may occur even in patients with globally preserved cognitive scores, indicating that these deficits can constitute an independent and clinically significant domain. Our findings highlight that lower scores on the MoCA, higher levels of depression, and lower educational attainment are key predictors of diminished social cognitive performance.

Key predictors of SCI also included age, executive dysfunction, and impulsivity. These results underscore the clinical importance of incorporating SCI evaluations into routine assessments for pwMS, both to enhance diagnostic accuracy and to inform personalized rehabilitation strategies aimed at improving social functioning. Future studies involving larger, longitudinally followed cohorts—and incorporating assessments of fatigue, general intelligence, and neuroimaging markers—are warranted to better elucidate the progression of SCI and to support the development of accessible, ecologically valid screening tools for widespread clinical use. Comprehensive neuropsychological evaluation should be considered a standard component of MS care to help mitigate the risk of social withdrawal and optimize patient outcomes.

## Author Contributions

**Ozlem Totuk**: conceptualization, investigation, writing–original draft, methodology, validation, writing–review and editing, formal analysis, project administration, data curation, resources, supervision. **Merve Turkkol**: conceptualization, investigation, methodology, validation, formal analysis, data curation, supervision. **Ebru Hatun Uludaşdemir**: conceptualization, investigation, methodology, validation, formal analysis, supervision. **Hasan Can Güdek**: conceptualization, investigation, methodology, validation, formal analysis, data curation, supervision. **Guldeniz Cetin erci**: writing–review and editing, conceptualization, investigation, methodology, validation, formal analysis, data curation, supervision. **Ipek Gungor dogan**: conceptualization, investigation, methodology, validation, data curation, supervision. **Damla Cetinkaya Tezer**: conceptualization, investigation, methodology, validation, data curation, supervision. **Sevki Sahin**: conceptualization, investigation, writing–review and editing, methodology, validation, formal analysis, data curation, supervision. **Serkan Demir**: conceptualization, investigation, methodology, validation, data curation, supervision.

## Conflicts of Interest

The authors declare no conflicts of interest.

## Peer Review

The peer review history for this article is available at https://publons.com/publon/10.1002/brb3.70691.

## Data Availability

The data that support the findings of this study are available on request from the corresponding author. The data are not publicly available due to privacy or ethical restrictions.
